# Exposure to residual concentrations of elements from a remediated coal fly ash spill does not adversely influence stress and immune responses of nestling tree swallows

**DOI:** 10.1093/conphys/cou018

**Published:** 2014-05-28

**Authors:** Michelle L. Beck, William A. Hopkins, John J. Hallagan, Brian P. Jackson, Dana M. Hawley

**Affiliations:** 1 Department of Fish and Wildlife Conservation, Virginia Tech, 106 Cheatham Hall, Blacksburg, VA 24061-0321, USA; 2 Department of Earth Sciences, Dartmouth College, 6105 Fairchild Hall, Hanover, NH 03755, USA; 3 Department of Biology, Virginia Tech, 2125 Derring Hall, Blacksburg, VA 24061-0406, USA

**Keywords:** bactericidal capacity, cell-mediated immunity, element, stress response, tree swallow

## Abstract

Anthropogenic activities often produce pollutants that can affect the physiology, growth and reproductive success of wildlife. Many metals and trace elements play important roles in physiological processes, and exposure to even moderately elevated concentrations of essential and non-essential elements could have subtle effects on physiology, particularly during development. We examined the effects of exposure to a number of elements from a coal fly ash spill that occurred in December 2008 and has since been remediated on the stress and immune responses of nestling tree swallows. We found that nestlings at the site of the spill had significantly greater blood concentrations of Cu, Hg, Se and Zn in 2011, but greater concentrations only of Se in 2012, in comparison to reference colonies. The concentrations of elements were below levels of significant toxicological concern in both years. In 2011, we found no relationship between exposure to elements associated with the spill and basal or stress-induced corticosterone concentrations in nestlings. In 2012, we found that Se exposure was not associated with cell-mediated immunity based on the response to phytohaemagglutinin injection. However, the bactericidal capacity of nestling plasma had a positive but weak association with blood Se concentrations, and this association was stronger at the spill site. Our results indicate that exposure to these low concentrations of elements had few effects on nestling endocrine and immune physiology. The long-term health consequences of low-level exposure to elements and of exposure to greater element concentrations in avian species require additional study.

## Abstract (precis)

We examined the effects of elements from a remediated fly ash spill on nestling tree swallow physiology. Nestlings were exposed to modestly elevated concentrations of elements including selenium near the spill. Bacteria killing capacity was positively related to selenium exposure, but element exposure was unrelated to other aspects of physiology.

## Introduction

Humans are rapidly altering the environment and while these changes may threaten the persistence of species and populations (Vitousek *et al.*, 1997), they may also have subtle, non-lethal effects on individuals. Exposure to anthropogenic pollutants can affect physiology (Acevedo-Whitehouse and Duffus, 2009; Martin *et al.*, 2010), compromise reproductive performance (Heinz, 1996; Baos *et al.*, 2012) and affect development of vertebrates (Markman *et al.*, 2011). Elements, including heavy metals, metalloids and trace elements, are one form of pollution that wildlife are exposed to through a number of anthropogenic processes, such as intensive agriculture (Ohlendorf *et al.*, 1986; Orłowski *et al.*, 2010), mining (Weech *et al.*, 2012), coal combustion (Rowe *et al.*, 2002) and metal smelting (Janssens *et al.*, 2001). Some of these elements are of known toxicological importance, such as arsenic (As), lead (Pb) and mercury (Hg), as well as elements that are nutritionally important but become toxic at elevated concentrations, such as copper (Cu), iron (Fe), selenium (Se) and zinc (Zn). At optimal dietary concentrations, the latter elements affect a variety of physiological processes, acting as enzyme cofactors and antioxidants and enhancing the immune response (Reilly, 2006; Wintergerst *et al.*, 2007). However, at higher concentrations they can cause oxidative stress (Janz *et al.*, 2010; Koivula and Eeva, 2010), increase susceptibility to infection (Sherman, 1992; Wintergerst *et al.*, 2007) and inhibit reproductive performance (Baos *et al.*, 2012). Given the important physiological functions of elements, individuals may be more sensitive to element contamination during early development, when exposure could permanently alter some physiological processes (Nyholm, 1998; Baos *et al.*, 2006a). Thus, exposure to even moderately elevated concentrations of some elements during development may affect physiological processes, such as stress and immune responses, which are directly relevant to survival and reproduction.

The stress response is one aspect of physiology that may be affected by exposure to elements. This response is regulated by the hypothalamic–pituitary–adrenal axis (HPA axis), which controls the release of glucocorticoids in vertebrates (Wingfield and Romero, 2001). Basal glucocorticoid concentrations are responsible for regulating energy balance, blood glucose and fatty acid levels (reviewed by Landys *et al.*, 2006), while stress-induced glucocorticoids are released in response to unexpected challenges and cause changes in behaviour and physiology that enhance the probability of survival (reviewed by Wingfield *et al.*, 1998; Sapolsky *et al.*, 2000). Exposure to elements can potentially affect the regulation of glucocorticoids via an array of mechanisms acting on different levels of the HPA axis. For example, release of glucocorticoids can be affected by altering the release of, or response to, corticotrophin-releasing hormone or adrenocorticotrophic hormone from the hypothalamus and pituitary, respectively (Potmis *et al.*, 1993; Handy, 2003; Gagnon *et al.*, 2006). Elements such as Cu, Fe, Mn and Zn are also important components of many enzymes (Marmiroli and Maestri, 2008), and increases in their concentrations above normal dietary levels could potentially affect enzymes associated with glucocorticoid metabolism (Hopkins *et al.*, 1997).

Given that elements have the potential to influence the production, release and clearance of glucocorticoids, it is not surprising that studies examining the effects of elements on basal and stress-induced glucocorticoid concentrations have produced mixed results. For example, in some avian studies, Hg exposure may reduce (Franceschini *et al.*, 2009; Herring *et al.*, 2012) or elevate basal glucocorticoid concentrations (Wada *et al.*, 2009). Basal glucocorticoid concentrations have not been significantly associated with mixtures of elements, including cadmium (Cd), Hg and Se [common eiders (*Somateria mollissima*), Wayland *et al.*, 2003], As, Cd, Cu, Pb and Zn [white storks (*Ciconia ciconia*), Baos *et al.*, 2006a], or As, Cu, nickel (Ni), Pb and Zn [pied flycatchers (*Ficedula hypoleuca*), Eeva *et al.*, 2005], but Cd, Se and Hg may influence basal glucocorticoids in interactions with other elements or body condition [lesser scaup (*Aythya affinis*), Pollock and Machin, 2009]. Effects on induced glucocorticoid concentrations produced using standardized handling restraint are also variable. Exposure to elements including Pb and Cd can enhance the stress-induced release of glucocorticoids (Wayland *et al.*, 2002; Baos *et al.*, 2006a), while exposure to Hg and Se can inhibit it (Wayland *et al.*, 2002; Wada *et al.*, 2009). In these same or very similar studies, other elements, including As, Cd, Hg, Se and Zn, appeared to have no effect on stress-induced glucocorticoids (Wayland *et al.*, 2002, 2003; Baos *et al.*, 2006a). Given that animals may be exposed to a variety of elements that may interact in complex ways (Marmiroli and Maestri, 2008; Zwolak and Zaporowska, 2012), it is important to understand how elements commonly found in the environment influence the HPA axis as well as other physiological processes.

Exposure to elements can also affect immune responses and disease resistance. Many immune responses are affected by nutritional condition (Alonso-Alvarez and Tella, 2001; Lifjeld *et al.*, 2002; Ponton *et al.*, 2013), and exposure to high concentrations of elements may reduce an individual's nutritional condition and hence their immune response (Ritchie *et al.*, 1994; Massányi *et al.*, 1999). Element exposure may directly affect the immune system by impairing immune cell function, altering protein synthesis, or through cytotoxic effects on immune organs (Koller, 1973; Blakley *et al.*, 1980; Dan *et al.*, 2000). Exposure to elements such as Pb increases disease prevalence in house sparrows (*Passer domesticus*, Bichet *et al.*, 2013), providing indirect evidence that element exposure impairs immunity. Exposure to elevated concentrations of Pb or a mixture of elements including As, Cd, Cu, Pb, Hg, Se and Zn was associated with reduced humoral immunity [zebra finch (*Taeniopygia guttata*) and great tit (*Parus major*), Snoeijs *et al.*, 2004, 2005], while cell-mediated immunity can be impaired by exposure to Hg in tree swallows (*Tachycineta bicolor*, Hawley *et al.*, 2009). However, other studies have detected no effect of these same elements on humoral (Wayland *et al.*, 2003; Biser *et al.*, 2004; Hawley *et al.*, 2009) or cell-mediated immunity (Snoeijs *et al.*, 2005; Baos *et al.*, 2006b), and a few studies have even detected stimulatory effects of Se exposure on these immune responses (Wayland *et al.*, 2002; Surai, 2006; Brady *et al.*, 2013). Robust innate and cell-mediated immune responses require adequate dietary concentrations of Cu, Fe, Se and Zn (reviewed by Maggini *et al.*, 2007; Wintergerst *et al.*, 2007), and it is possible that moderate increases in these elements, below levels associated with toxic effects, are responsible for the enhanced immune responses detected in some studies.

We examined the effects of environmental exposure to a mixture of elements from a recently remediated coal fly ash spill on the stress and immune responses of nestling tree swallows. Coal fly ash is one of the largest solid waste streams produced globally and represents a significant source of an array of elements to aquatic systems (Rowe *et al.*, 2002; NRC, 2006). Fly ash spills, such as the one that took place at our study site, represent extreme circumstances, but aquatic disposal of fly ash continually introduces elements into streams and rivers around the world. Fly ash contains elevated concentrations of several elements including As, Hg, Se, V and others that pose health risks to humans and wildlife (Rowe *et al.*, 2002; NRC, 2006). At the time of our study, remediation efforts at our site were largely completed and the concentrations of elements in this system were below levels associated with negative effects on reproduction or survival in many avian species (Ohlendorf, 2003; Dauwe *et al.*, 2005; Brasso and Cristol, 2008). However, we hypothesized that exposure during development to low concentrations of elements associated with coal fly ash would affect avian physiology. In light of the discrepancies amongst study systems highlighted above, we focused on the relationship between element exposure and the HPA axis and immune responses in an effort to contribute to this growing body of literature.

## Methods

### Study species

Tree swallows are one of the primary model species used to address the movement of contaminants from aquatic to terrestrial ecosystems (Custer, 2011) and are used extensively in field studies of physiology, life history and behaviour (Robertson *et al.*, 2011). Tree swallows are aerial insectivores and, when breeding in riparian areas, feed primarily on emerging aquatic insects (Custer *et al.*, 2010; Custer, 2011; Beck *et al.*, 2013). A few studies have examined the effects of one element, Hg, on the stress and immune responses of tree swallows. Some of these studies have detected reduced basal corticosterone concentrations with greater Hg exposure (Franceschini *et al.*, 2009) and others increased basal but reduced stress-induced corticosterone concentrations with greater Hg exposure (Wada *et al.*, 2009). The cell-mediated immune response was negatively affected by exposure to Hg, but Hg exposure did not affect the humoral immune response (Hawley *et al.*, 2009). Although these studies demonstrated that Hg exposure related to aspects of tree swallow physiology, no studies have examined the effects of many of the elements found in fly ash on the immune or stress responses of tree swallows.

### Study site

In December 2008, a coal fly ash impoundment at the Tennessee Valley Authority fossil plant in Kingston, TN, USA (35.8722°N, 84.5250°W) ruptured, releasing 4.1 million m^3^ of coal fly ash slurry into the Emory River, which then flowed into the Clinch and Tennessee Rivers (TVA, 2009). In the 2.5 years following the spill, most of the coal fly ash was removed from the river system but ∼400 000 m^3^ remained at the time of our study (TVA, 2011a).

**Figure 1:**
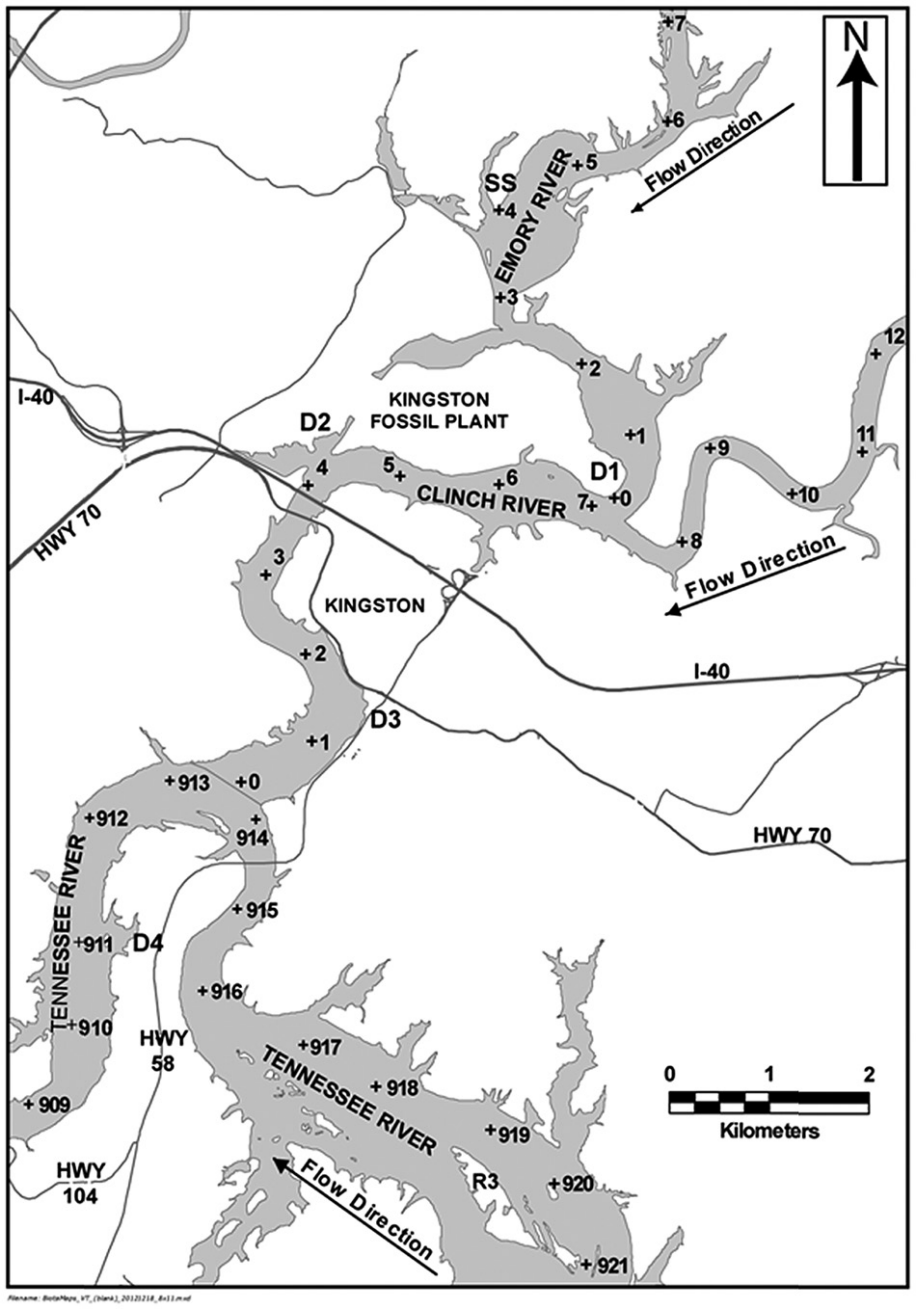
Tree swallow colonies located near Kingston, TN, USA. The study area consisted of two highly impacted colonies located on the Emory River. One was located at the site of the spill (spill site, SS, *n* = 94) and the second at the confluence of the Clinch and Emory River (downstream 1, D1, *n* = 31) 4 km downstream from the spill. Two moderately impacted colonies were located on the Clinch River at downstream 2 (D2, *n* = 31) and downstream 3 (D3, *n* = 43) and were 3.0 and 7.0 km, respectively, downstream from the confluence with the Clinch River. A low-impacted colony was located downstream on the Tennessee River (D4, *n* = 51) 2.5 km from the confluence with the Clinch and Tennessee Rivers. We used three reference colonies; two were located near Lenoir City, TN, USA 30.5 km east of Kingston. Reference 1 (R1, *n* = 46) was located at Ft Loudoun Dam on the Tennessee River and reference 2 (R2, *n* = 53) at Tellico Dam on the Little Tennessee River. Reference 3 was located on Long Island (R3, *n* = 53) on the Tennessee River 5.5 km upstream from the confluence with the Clinch River. We also placed boxes at Melton Hill Dam (MD, *n* = 68) on the Clinch River, which served a role analogous to a positive control. The sites MD, R1 and R2 are not pictured here. *n* refers to the number of nest boxes located at each colony. River kilometres are given in each river.

We studied tree swallows along an element contamination gradient and at several reference colonies in Roane and Loudon Counties, TN, USA, from May to July 2011 and 2012 (Fig. 1). We placed nest boxes at the spill site (SS) and at four colonies located downstream from the spill site (hereafter ‘downstream’, D1–D4). Colony D1 was located ∼3.5 km by river from the spill site, while D4 was located ∼14 km by river from the spill site. We had three reference colonies; two located ∼30.5 km east of Kingston at Ft Loudoun Dam (Reference 1) and at Tellico Dam (Reference 2), while Reference 3 (R3) was located on the Tennessee River ∼6 km by river upstream from the confluence with the Clinch River, at Long Island. We also placed boxes at Melton Hill Dam (MD) on the Clinch River, which served a role analogous to a positive control because preliminary data gathered prior to this study indicated that tree swallows are exposed to ash-related contaminants such as Se at this colony (ARCADIS, 2011). The source(s) of this contamination is unclear, but could include the Bull Run Fossil Plant (Stantec, 2009; TVA, 2011b), a former coal ash storage pond associated with the Y-12 Security Complex (Cook *et al.*, 1999), or other non-point source pollution (USDA, 2009). We grouped these colonies into four types, the spill site, all downstream colonies (D1–D4), all reference colonies (R1–R3) and Melton Hill (MD), based on how they were impacted by the fly ash spill. By sampling nestlings from these colonies, we were able to examine the physiological responses of nestlings across a range of low to moderately elevated element concentrations.

We placed clean nest boxes in each area when tree swallows were arriving at the breeding grounds and prospecting for nest sites. All of the colonies were established at least 1 year prior to this study except for D1, which we established in 2011. Nest boxes were 25 cm × 20 cm × 41 cm with a 2.5 cm entrance hole and were mounted 1.5 m above the ground on metal conduit. All entrances were oriented toward the water, and boxes were located within 70 m of the shore to facilitate foraging on emerging aquatic insects. We spaced nest boxes 15 m apart when in a single row, or 20 m apart with a staggered alignment of two or more rows. We checked nest boxes every 4 days, beginning in late March, for signs of nesting activity and to obtain basic reproductive data. When nestlings were 13 days old, we banded them and obtained blood samples for the corticosterone assay, immune challenge or element analysis (see below). For each nestling, we measured the length of the left and right tarsus (each tarsus was measured twice) and body mass.

### Response to handling stress

In 2011, we examined the effect of element exposure on the stress response of 13-day-old nestlings by subjecting them to a standardized handling stress protocol (after Wingfield and Romero, 2001). We obtained a blood sample (∼60 μl) from up to half of the nestlings in a brood within 3 min of disturbing the box. These nestlings were then held alone in a cloth bag for 30 min, after which a second blood sample was obtained. Samples were stored in a cooler containing ice blocks before being centrifuged for 5 min at 9783 *g*, and the plasma fraction was removed and stored at −20°C in the field-house and stored long-term at −80°C.

In the autumn of 2011, we randomly selected plasma from one nestling from each nest that we subjected to the handling stress protocol and quantified basal and induced corticosterone concentrations in 125 nestlings across all colonies. By using a single sample per nest, we avoided issues with pseudoreplication. We used Enzo Life Sciences enzyme immunoassay kits (catalogue no. 901-097) using a procedure previously validated for tree swallows by Wada *et al.* (2009). We haphazardly distributed samples from different colonies equally across 10 96-well plates. We diluted 12 μl of plasma with an equal volume of 3% steroid displacement buffer and then diluted samples 1:20 with assay buffer. On each plate, a standard curve that ranged from 15.6 to 2000 pg/ml was run in triplicate. A 500 pg/ml corticosterone standard was also run in triplicate on each plate, and each plasma sample was run in duplicate. The assay had a detection limit of 1.1 ng/ml, and any samples (*n* = 49) that fell below this were assigned half of the detection limit for their corticosterone concentration. Samples that fell below the detection limit were equally distributed among Melton Hill, the spill site, downstream and reference colonies (χ^2^ = 3.04, d.f. = 3, *P* = 0.385) and assays (χ^2^ = 8.55, d.f. = 7, *P* = 0.287). Three nestlings had induced corticosterone concentrations that were below the detection limit, and we ran statistical tests including and excluding these samples. We calculated intra-assay variation as the average coefficient of variation between duplicate samples on each plate and inter-assay variation as the coefficient of variation among the standards on every plate. Intra-assay variation was 11.2% and inter-assay variation 13.3%.

### Immune response

We examined the effects of elements on aspects of the immune response in nestling tree swallows in June and July 2012. We randomly selected a single nestling from each nest to avoid issues with pseudoreplication. We quantified the response of 37 13- to 14-day-old tree swallow nestlings to phytohaemagglutinin (PHA; Sigma Aldrich, St Louis, MO, USA) by injecting the patagium (wing web) of nestlings with 0.15 mg of PHA dissolved in 30 μl of phosphate-buffered saline (PBS; after Smits *et al.*, 1999). The injection of PHA leads to a localized swelling due to the influx and proliferation of T cells and leukocytes at the injection site (Martin *et al.*, 2006) and a build-up of free radicals that are produced during phagocytosis by components of the innate immune system (Peretz, 1989). Feathers were first cleared from the wing web, and the area was sterilized using 70% ethanol. One individual held the nestling with its right wing extended in a standardized position, while a second individual (J.J.H.) made all measurements and performed the injections. Prior to and 24 h following injection, the thickness of the wing web was quantified to the nearest 0.01 mm using a micrometer. To avoid bias, the micrometer dial was not visible to the measurer while making the measurement. We made five pre- and five post-injection measurements at the injection point, discarded the lowest and highest values in each set and used the remaining three values to produce average pre- and post-injection thicknesses. We divided the difference between the post- and pre-injection measurements by the pre-injection measurement and multiplied this value by 100 to calculate the percentage increase in swelling caused by the injection and used this as our measure of the cell-mediated immune response.

We examined innate immunity in nestlings by evaluating the bactericidal capacity of plasma (Liebl and Martin, 2009). Blood samples were obtained from 96 nestlings, one from each nest, 13 days post-hatch. The area around the puncture site was cleansed with 70% ethanol prior to blood collection from the brachial vein. We collected 120 μl of blood in heparinized capillary tubes; 60 μl was reserved for element analysis and the other 60 μl used to assess bactericidal capacity. Samples were stored in coolers with ice packs in the field (≤4 h) and were refrigerated prior to being centrifuged (≤2 h) at 9783 *g* for 5 min for the bactericidal assay. The plasma fraction was placed in a sterile 0.5 ml tube and refrigerated until all samples gathered that day were centrifuged. All samples were run on the day of collection in order to minimize degradation. The majority of samples were run in triplicate and occasionally duplicate (*n* = 2) by diluting 3.5 μl of plasma with 31.5 μl of sterile PBS (1:10 dilution). We added 12.5 μl of 10^5^ bacteria/ml *Escherichia coli* solution (ATCC 8739, E^power^ microorganisms; Microbiologics^®^, St Cloud, MN, USA) to each tube and vortexed each sample. Samples were incubated at 37°C for 30 min, then 250 μl of tryptic soy broth (TSB; Sigma Aldrich ) was added to each tube, and samples were incubated for an additional 12 h at 37°C. Positive controls were prepared in triplicate by adding 12.5 μl of 10^5^ bacteria/ml *E. coli* solution to 250 μl of TSB, and we prepared duplicate blanks by combining 50 μl of PBS with 250 μl of TSB. We prepared an additional control in duplicate that contained 3.5 μl of plasma, 250 μl of TSB and 50 μl of PBS to check that bacteria were not introduced during bleeding or sample processing.

Following the 12 h incubation, samples were vortexed, and a Nanodrop Spectrophotometer (ND-2000; Thermo Scientific, Pittsburgh, PA, USA) was used to measure the absorbance of each sample at an optical density of 300 nm (Liebl and Martin, 2009). The absorbance of each sample and the positive controls were each averaged and used to calculate the proportion of bacteria killed as one minus (average sample absorbance/average positive control absorbance). The Nanodrop arm was cleansed between each sample with 70% ethanol, and the entire work area was cleansed with ethanol before and after each work day.

### Analyses of elements

Blood samples from nestlings were shipped overnight on dry ice to the Trace Element Analysis Core at Dartmouth College (Hanover, NH, USA). Concentrations of As, Ba, Cd, Cr, Cu, Fe, Mn, Hg, Se, Sr, Tl, V and Zn present in blood were quantified for each sample using inductively coupled mass spectrometry following EPA method 6020A (EPA, 2008). Samples were digested using an open vessel acid digestion with 0.5 ml of 9:1 HNO_3_:HCl (Optima, Fisher Scientific, St Louis, MO, USA) using microwave heating at 105°C for 45 min. After cooling, 0.1 ml H_2_O_2_ was added to the samples and they were taken through a second heating step (adpated from EPA, 1996). The samples were then diluted to 10 ml with deionized water. Digested samples were analysed for element concentrations by collision cell inductively coupled mass spectrometry (7700x; Agilent, Santa Clara, CA, USA). Concentrations of As, Ba, Cd, Cr, Cu, Fe, Mn, Sr, Tl, V and Zn (He collision mode), Se (reaction mode) and Hg (normal mode) were quantified in each sample. Digestion quality control measures included digestion blanks, fortified blanks and reference materials at a frequency of one each per 20 samples. There was insufficient blood to allow for digestion of duplicates or spikes. Analytical sample duplicates and spikes were performed at a frequency of one each per 20 samples. Additional quality control consisted of reporting limit checks, interference checks and initial and continuing calibration checks and blanks.

Arsenic, Cd, Cr, Tl and V concentrations were below detection limits (BDL) in over half of the nestling blood samples from all colonies in both years and were not considered further (Table 1). In 2011, Mn concentrations were BDL and in 2012, Hg concentrations were BDL in over half of the samples from each colony and were excluded from analyses in those years. The average relative percentage difference for eight elements over five analysis duplicates was 12 ± 2%. The average percentage recovery for 13 elements over five analysis spiked samples was 97 ± 21%. The average percentage recovery for As, Cd, Cu, Fe, Mn, Hg, Se, Sr and Zn was 100 ± 13% for five separate digestions of the standard reference material NIST 2976. Digestion blanks were less than reporting limits and fortified spike recoveries were generally 90–110% throughout the digestion batches. Other elements were not certified in the NIST standard.

**Table 1:** Mean values and standard errors for blood element concentrations (in micrograms per gram wet mass) in nestling tree swallows among colonies in 2011 and 2012

Element	Reference	Melton Hill	Spill site	Downstream	*F*	*P* value	Average detection limit
Ba 2011	0.74 ± 0.07	0.94 ± 0.09	0.94 ± 0.08	0.79 ± 0.05	2.15	0.10	0.049
2012	0.90 ± 0.08	0.61 ± 0.09	0.65 ± 0.08	0.69 ± 0.07	0.98	0.41	0.015
Cu 2011	0.28 ± 0.01	0.31 ± 0.02	0.35 ± 0.01	0.29 ± 0.01	6.20	0.001	0.080
2012	0.42 ± 0.07	0.32 ± 0.07	0.29 ± 0.07	0.39 ± 0.05	2.10	0.11	0.044
Fe 2011	367.7 ± 11.9	380.2 ± 14.6	393.5 ± 13.3	349.0 ± 9.1	2.99	0.03	8.22
2012	481.3 ± 34.7	405.2 ± 38.3	353.4 ± 33.9	379.7 ± 28.3	2.69	0.05	1.47
Mn 2011	BDL	BDL	BDL	BDL	NA	NA	0.068
2012	0.065 ± 0.007	0.056 ± 0.008	0.040 ± 0.007	0.046 ± 0.006	3.00	0.04	0.015
Hg 2011	0.013 ± 0.003	0.008 ± 0.004	0.014 ± 0.003	0.010 ± 0.002	5.69	0.001	0.029
2012	BDL	BDL	BDL	BDL	NA	NA	0.029
Se 2011	0.85 ± 0.11	2.65 ± 0.14	1.79 ± 0.13	0.99 ± 0.09	29.45	<0.001	0.312
2012	0.98 ± 0.11	0.88 ± 0.12	1.74 ± 0.11	1.05 ± 0.09	14.11	<0.001	0.012
Sr 2011	0.094 ± 0.020	0.071 ± 0.024	0.096 ± 0.022	0.116 ± 0.015	2.06	0.11	0.020
2012	0.069 ± 0.007	0.049 ± 0.007	0.068 ± 0.007	0.051 ± 0.005	2.75	0.05	0.037
Zn 2011	6.21 ± 0.20	5.68 ± 0.24	6.64 ± 0.22	5.54 ± 0.15	6.26	0.001	2.17
2012	8.34 ± 0.61	6.11 ± 0.68	5.73 ± 0.60	6.63 ± 0.50	5.73	0.001	1.47

Concentrations of several elements were below the detection limit (2011 detection limit/2012 detection limit) in both years and were not considered further, as follows: As (0.009/0.006), Cd (0.009/0.007), Cr (0.098/0.088), Tl (0.009/0.001) and V (0.016/0.015). Number of nests sampled at each colony, 2011: reference = 30, Melton Hill Dam = 20, spill site = 24 and downstream = 51; and 2012: reference = 22, Melton Hill Dam = 18, spill site = 23, and downstream = 33 (2011 d.f. = 3, 121; 2012 d.f. = 3, 92). Abbreviations: BDL, below detection limit; and NA, not assessed.

### Statistical analysis

We used Kolmogorov–Smirnov tests and normality plots to determine whether variables met the assumptions of parametric tests. Element concentrations and basal and induced corticosterone concentrations were not normally distributed and were log transformed prior to analysis, which successfully normalized the data. We first compared element concentrations among colony types (reference, spill site, downstream and Melton Hill) using a MANOVA followed by univariate ANOVAs and Tukey's tests to determine which elements were significantly elevated in the system due to the fly ash spill. Only elements that were found at the spill site at significantly higher concentrations than all reference colonies were included in the analysis that examined the effects of element exposure on the stress and immune responses of nestlings. Given that Hg concentrations were BDL in 2012 and Mn concentrations were BDL in 2011, we made these comparisons separately for each year. We calculated body condition for nestlings as the residuals of a regression of mass on tarsus length (*r*^2^ = 0.243, d.f. = 124, *P* < 0.001**)**. While the use of residuals as a measure of body condition is controversial (Green, 2001), studies have shown that residuals do correlate well with lipid reserves (Ardia, 2005; Schulte-Hostedde *et al.*, 2005) and other studies have shown that residual body mass is related to the immune and the stress responses in avian species (Pollock and Machin, 2009; Palacios *et al.*, 2012).

We used linear regressions and backward elimination of non-significant terms to examine the effects of clutch initiation date, element concentrations, body condition and two-way interactions between condition and element concentrations on the immune and stress responses of nestlings. We eliminated non-significant interaction terms first, followed by main effects that were not significantly related to physiology. We allowed terms to remain in the model as long as *P* ≤ 0.10, but considered their contribution to be statistically significant only when *P* ≤ 0.05. For the stress response, we performed separate regressions with basal and induced corticosterone as the dependent variables because these two parameters represent distinct physiological responses that engage different receptor types (Kloet, 1991). To examine the immune responses, we used the percentage of bacteria killed and wing web swelling as dependent variables. Given that much of the variance in element concentrations, particularly for Se, was attributable to variation at the spill site, we ran an additional iteration of any statistically significant model that focused only on samples collected at this colony. Given that element concentrations and colony type were confounded in the analysis, we did not include colony type in the regression models. Rather, we also compared corticosterone concentrations and immune responses among colony types using an ANCOVA, with clutch initiation date included as a covariate. All statistical tests were two-tailed, with α = 0.05. All statistical analyses were performed using PASW 18 (SPSS, 2009).

## Results

### Concentrations of elements among colonies

We first compared concentrations of elements among the four colony types, i.e. reference, the spill site, downstream and Melton Hill. In 2011, we found that concentrations of Ba and Sr did not differ significantly among colony types (Table 1; all *P* ≥ 0.10). However, we found that Cu, Fe, Hg, Se and Zn concentrations differed significantly among colonies, and *post hoc* tests indicated that the spill site had greater concentrations than the reference colonies for all of these elements (*P* ≤ 0.001) except for Fe (*P* = 0.47). Given that only Cu, Hg, Se and Zn were significantly elevated at the spill site in comparison to reference colonies, we focused on the effect of these elements on the basal and induced plasma corticosterone concentrations of nestlings. Copper, Se and Zn concentrations were not significantly correlated with each other (all *r* ≤ 0.11, all *P* ≥ 0.23), but Hg concentrations correlated positively with Cu (*r* = 0.27, *P* = 0.002) and were almost significantly correlated with Se concentrations (*r* = 0.16, *P* = 0.07). In order to reduce the number of tests performed while avoiding issues with collinearity, we performed two separate backward elimination regressions with different combinations of the elements, one that included Cu, Se and Zn and a second that used only Hg.

In 2012, we found that concentrations of Ba and Cu did not differ significantly among colony types (Table 1; all *P* ≥ 0.06). Concentrations of Fe, Mn, Sr and Zn differed significantly among colony types, but not in ways that indicated an association with the fly ash spill. *Post hoc* tests indicated that concentrations of Fe, Mn, Sr and Zn were significantly greater at reference colonies than those at the spill site (all *P* ≤ 0.05). Only concentrations of Se remained significantly elevated at the spill site in comparison to reference colonies (*P* < 0.001). Thus, all of the immune challenge analyses focused on nestling blood Se concentrations.

### Stress response

In 2011, we examined the effect of element exposure on the stress response of nestling tree swallows. Basal corticosterone concentrations averaged 2.7 ± 0.24 ng/ml (range 0.57–17.4 ng/ml) and induced corticosterone concentrations averaged 11.8 ± 0.87 ng/ml (range 0.57–62.1 ng/ml) in all of the colonies combined. Basal corticosterone concentrations differed significantly among colony types (Table 2; *F*_3,120_ = 3.6, *P* = 0.02), and Tukey's *post hoc* tests indicated that basal corticosterone concentrations were significantly greater at downstream colonies than at reference colonies. Induced corticosterone concentrations also differed significantly among colony types (Table 2; *F*_3,117_ = 3.7, *P* = 0.01), and this result did not change when the three individuals with induced corticosterone concentrations below the assay detection limit were included in the analysis (*F*_3,120_ = 4.7, *P* = 0.004). Induced corticosterone concentrations were significantly lower at downstream colonies than at reference colonies or at the spill site (both *P* ≤ 0.04). Element concentrations and their interaction with condition were unrelated to basal corticosterone concentrations (Table 3; full model with Cu, Se and Zn, *r*^2^ = −0.07, d.f. = 116, *P* = 0.37; full model with Hg, *r*^2^ = −0.06, d.f. = 120, *P* = 0.13). The only term that remained in the final version of these models was nestling condition, which had a very weak but statistically significant negative relationship with basal corticosterone concentrations (final models, *r*^2^ = −0.03, d.f. = 123, *P* = 0.05). Likewise, induced corticosterone concentrations were unrelated to element exposure and the interactions between condition and element exposure (Table 3; full model with Cu, Se and Zn, *r*^2^ = 0.09, d.f. = 113, *P* = 0.19; full model with Hg, *r*^2^ = −0.07, d.f. = 117, *P* = 0.06), and this did not change if the three nestlings with induced corticosterone concentrations below the assay detection limit were included in the analysis for the model including Cu, Se and Zn (*r*^2^ = 0.10, d.f. = 116, *P* = 0.14). While the full model including Hg was statistically significant when these three individuals were included (*r*^2^ = −0.09, d.f. = 120, *P* = 0.03), this was caused by an association between induced corticosterone concentrations and measurement date rather than Hg exposure (Table 3). For both groups of elements, clutch initiation date remained in the final models and had a weak, negative relationship with induced corticosterone concentrations (Table 3; final models, *r*^2^ = −0.05, d.f. = 123, *P* = 0.01), and including or excluding the individuals with induced corticosterone below the assay detection limit did not influence this relationship.

**Table 2:** Mean values and standard errors for stress and immune responses in nestling tree swallows among colonies

Response	Reference	Melton Hill	Spill site	Downstream	*F*	*P* value
Basal corticosterone (ng/ml)	1.80 ± 0.47	2.25 ± 0.58	1.99 ± 0.52	3.69 ± 0.36	3.6	0.02
Induced corticosterone (ng/ml)	16.12 ±1.7	9.64 ± 2.1	13.98 ± 1.9	9.10 ± 1.3	4.7	0.004
Corticosterone samples (*n*)	30	20	24	51		
PHA (%)	58.3 ± 15.5	78.9 ± 12.2	75.8 ± 9.6	72.5 ± 10.4	0.41	0.75
PHA samples (*n*)	5	8	13	11		
BKA (%)	17.2 ± 2.5	18.4 ± 2.7	17.3 ± 2.4	20.6 ± 2.0	0.55	0.65
BKA samples (*n*)	22	18	23	33		

The stress response was quantified in 2011, while the immune responses were quantified in 2012. Least-squares means are given for basal and induced corticosterone concentrations because Julian clutch initiation date had a significant influence on both the basal and induced corticosterone concentrations. Basal and induced corticosterone, d.f. = 3, 120; phytohaemagglutinin (PHA), d.f. = 3, 33; and bactericidal killing assay (BKA), d.f. = 3, 92. *n* refers to the number of nests sampled at each colony.

**Table 3:** Full and reduced model results from multiple regressions examining the effects of element exposure on the stress responses of nestling tree swallows

Term	β	*P* value
Basal corticosterone full model		
Intercept	0.864	0.12
Cu	−0.263	0.58
Se	−0.034	0.83
Zn	−0.345	0.40
Condition	−0.026	0.11
Clutch initiation date	−0.003	0.14
Cu × condition	0.085	0.53
Se × condition	−0.003	0.95
Zn × condition	0.077	0.62
Basal corticosterone full model Hg		
Intercept	0.533	0.28
Hg	−0.095	0.56
Condition	−0.029	0.07
Clutch initiation date	−0.003	0.14
Hg × condition	−0.024	0.74
Basal corticosterone final model both		
Intercept	0.191	<0.001
Condition	−0.032	0.04
Induced corticosterone full model		
Intercept	1.788	<0.001
Cu	−0.184	0.63
Se	0.121	0.33
Zn	−0.327	0.33
Condition	−0.003	0.85
Clutch initiation date	−0.004	0.03
Cu × condition	0.171	0.12
Se × condition	−0.021	0.58
Zn × condition	−0.075	0.56
Induced corticosterone full model Hg		
Intercept	1.630	<0.001
Hg	−0.041	0.75
Condition	−0.001	0.94
Clutch initiation date	−0.005	0.01
Hg × condition	0.098	0.10
Induced corticosterone final model both		
Intercept	1.700	<0.001
Clutch initiation date	−0.005	0.01

From the full model, we used backward elimination, beginning with interaction terms, to remove terms that did not contribute significantly to the model fit until only statistically significant terms remained. Basal and induced corticosterone models converged on the same final models (indicated by ‘both’ in the table) that included only nestling condition or clutch initiation date, respectively. For the analysis, induced corticosterone models exclude the three individuals with induced corticosterone concentrations below the assay detection limit; however, including these individuals produced nearly identical results.

### Immune response

**Figure 2:**
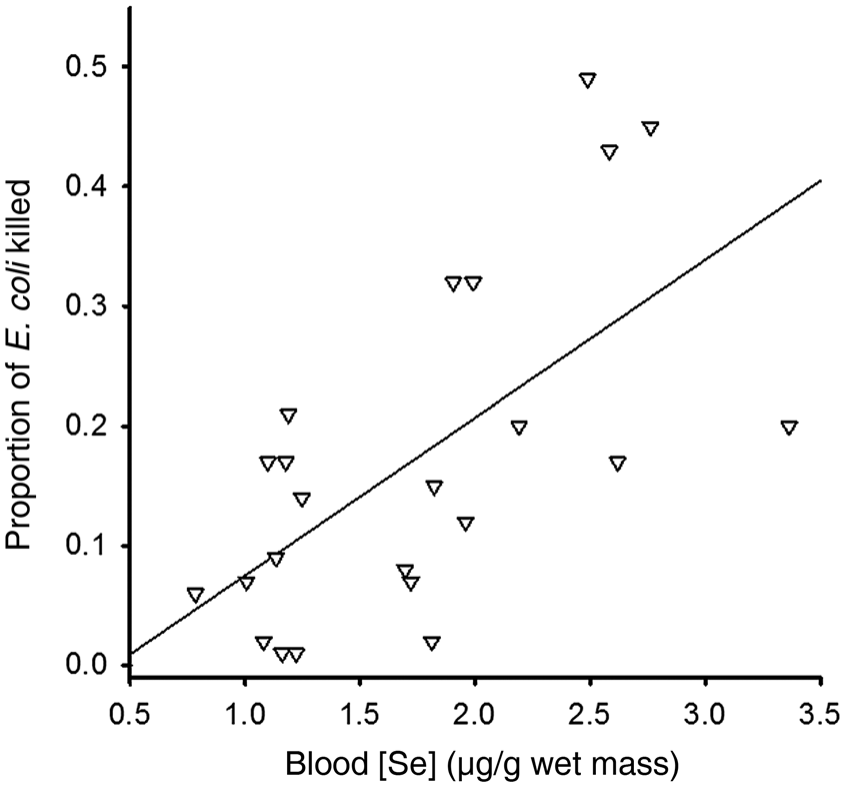
The relationship between Se exposure and bactericidal capacity in nestling tree swallows at the spill site. Higher Se concentrations were strongly associated with greater bactericidal capacity at this colony (*r*^2^ = 0.40, d.f. = 21, *P* = 0.001). Analyses were performed with log-transformed Se concentration, but we show untransformed data for clarity.

In 2012, we evaluated the effects of Se exposure on the cell-mediated and innate immune responses of nestling tree swallows. Among all of the colonies, the average response (percentage increase in swelling) to PHA injection was 73 ± 6% (range 17–160%), and we found no significant differences among colony types in the PHA-induced swelling (Table 2). The cell-mediated immune response of nestlings was not related to clutch initiation date, body condition, Se concentrations or any of the interaction terms (Table 4; full model *r*^2^ = −0.06, d.f. = 32, *P* = 0.75; final model *r*^2^ = −0.02, d.f. = 35, *P* = 0.45). The mean bactericidal capacity of nestling plasma was 18.7 ± 1.17% (range 10–49%), and we found no differences among colonies in bactericidal capacity (Table 2; *P* = 0.65). The bactericidal capacity of nestling plasma was not influenced by clutch initiation date, residual body mass or the interaction between Se exposure and condition, but was positively related to Se exposure (Table 4; full model *r*^2^ = 0.10, d.f. = 91, *P* = 0.05). Selenium concentrations remained in the final model and indicated a weak, but statistically significant positive relationship between blood Se concentrations and the bactericidal capacity of plasma (Table 4; final model, *r*^2^ = 0.07, d.f. = 95, *P* = 0.01). We ran this model again using data from the spill site alone and found a strong positive relationship between bactericidal capacity and Se concentrations (Fig. 2; final model, *r*^2^ = 0.40, d.f. = 21, *P* = 0.001). Our assay of cell-mediated immune response was unrelated to bactericidal capacity (*P* = 0.24).

**Table 4:** Full and reduced model results from multiple regressions examining the effects of selenium exposure on the immune responses of nestling tree swallows

Term	β	*P* value
PHA full model		
Intercept	−1.267	0.84
Se	−0.067	0.84
Condition	−0.017	0.56
Clutch initiation date	0.011	0.75
Se × condition	0.149	0.28
PHA final model		
Intercept	0.750	<0.001
Se	−0.220	0.45
BKA full model		
Intercept	0.038	0.84
Se	0.050	0.01
Condition	0.006	0.18
Clutch initiation date	0.001	0.62
Se × condition	−0.005	0.53
BKA final model		
Intercept	0.128	<0.001
Se	0.050	0.01
SS BKA full model		
Intercept	0.739	0.25
Se	0.430	0.02
Condition	−0.020	0.19
Clutch initiation date	−0.004	0.28
Se × condition	−0.092	0.35
SS BKA final model		
Intercept	0.062	0.12
Se	0.529	0.001

From the full model, we used backward elimination, beginning with interaction terms, to remove terms that did not contribute significantly to model fit. Abbreviations: BKA, bactericidal killing assay; PHA, phytohaemagglutinin; SS, spill site.

## Discussion

We examined the effects of exposure to elements from a recently remediated coal fly ash spill on the stress and immune responses of nestling tree swallows. We found that nestlings were exposed to elevated concentrations of some elements, particularly Se, at the spill site. However, we found no evidence of adverse effects on nestling physiology associated with the remediated fly ash spill. Element exposure was not related to the stress response of nestling tree swallows and was unrelated to a measure of the cell-mediated immune response. Only bactericidal capacity was affected by element exposure; greater exposure to Se was weakly associated with enhanced bactericidal capacity among all of the colonies, but strongly associated with Se exposure at the spill site. Overall, we suggest that remediation efforts and natural processes (e.g. offsite transport and dilution) in the years following the spill have left only modest concentrations of elements in the system and these low concentrations do not adversely affect the immune and stress physiology of swallows.

In 2011, we found that nestlings at the spill site were exposed to elevated concentrations of Cu, Hg, Se and Zn in comparison to nestlings in reference colonies, while in 2012, nestlings at the spill site were exposed to elevated Se only. Selenium is the primary driver of ecological risk in systems polluted by fly ash, and we found that even 4 years after the spill and massive remediation efforts, nestlings at the spill site were exposed to concentrations of Se elevated above those of reference colonies. However, most blood Se concentrations at the spill site were below concentrations typically associated with reduced survival or condition in avian species (Ohlendorf and Heinz, 2011). Blood Se concentration at the spill site ranged between 1.08 and 2.83 μg/g wet mass in 2011 and between 0.78 and 3.36 μg/g wet mass in 2012. Blood Se concentrations above 1.0 μg/g wet mass are considered a threshold level for concern (reviewed by Ohlendorf and Heinz, 2011), but many studies detect effects on adult survival or body mass only at blood Se concentrations above 5.0 μg/g wet mass (Heinz and Fitzgerald, 1993). However, blood Se concentrations within a range similar to those found in our study have been associated with reduced body mass in captive American kestrels (*Falco sparverius*) fed high-Se diets (Yamamoto and Santolo, 2000) and with signs of oxidative stress in emperor geese (*Chen canagica*, Franson *et al.*, 2002). All of these studies were conducted in laboratory animals that were exposed to Se in their diet for a minimum of 11 weeks (Heinz and Fitzgerald, 1993; Yamamoto and Santolo, 2000; Franson *et al.*, 2002). In our study, nestlings were exposed to elevated Se levels for only 13 days prior to sampling, and this may be why we found no negative physiological effects of these concentrations of Se when other researchers have.

Mercury, copper and zinc are other important elements found in some sources of fly ash, depending on the parent coal composition and combustion procedures used at power plants (Rowe *et al.*, 2002; NRC, 2006). In 2011 at the spill site, Hg concentrations in swallow blood ranged from 0.004 to 0.025 μg/g wet mass. These concentrations are one-14th of the concentrations that caused physiological effects in a study by Wada *et al.* (2009) at a highly Hg-contaminated site in Virginia. Indeed, our highest Hg concentration is comparable to those found in blood samples at the reference colonies, 0.017 μg/g wet mass, in the study by Wada *et al.* (2009). Concentrations of Cu and Zn at the spill site are largely similar to concentrations of these elements found in white storks, in studies that detected few effects of these elements on physiology (Baos *et al.*, 2006a, b).

Consistent with the low observed exposure to elements experienced by swallows in our remediated study system, we found no evidence of adverse physiological effects on the HPA axis. Basal and stress-induced corticosterone concentrations of swallows in this system were not related to element exposure. Indeed, corticosterone concentrations of swallows from impacted colonies were similar to the corticosterone concentrations found at reference sites in other studies (Franceschini *et al.*, 2008, 2009; Wada *et al.*, 2009). We did find subtle differences in basal and induced corticosterone concentrations among colony types, but not between the spill site and reference colonies, indicating these differences are not related to the fly ash spill. Downstream colonies had significantly higher basal corticosterone concentrations than reference colonies, and induced corticosterone concentrations were significantly higher at downstream colonies than those at the spill site and reference colonies. It is likely that the slight differences in corticosterone concentrations at downstream colonies are related to subtle ecological differences among colonies, such as resource availability, rather than contaminant exposure.

Likewise, we found few effects of elements from the fly ash spill on the immune response of nestling tree swallows. We found no differences among colonies in either the response to PHA injection or bactericidal capacity. We expected that blood Se concentration would affect the immune response, because adequate dietary concentrations of Se are necessary for robust innate and acquired immune responses (Maggini *et al.*, 2007; Wintergerst *et al.*, 2007). However, we found no relationship between the blood Se concentrations and the nestling response to PHA injection. Adult common eiders fed a high-Se diet had blood Se concentrations >8 μg/g wet mass and produced smaller swellings in response to PHA injection than control birds (Franson *et al.*, 2007). Thus, higher Se concentrations and more prolonged exposure than found in our study may influence this aspect of immunity. In contrast, we found that the bactericidal capacity of nestling plasma, an assay of innate immunity, showed a strong, positive relationship to their blood Se concentration at the spill site, and there was a weak association when all colonies were included in the analysis. The bacteria-killing capacity of plasma reflects several aspects of the innate immune response, including the ability of complement enzymes and lysozyme to destroy cell walls and lyse cell membranes of bacteria (reviewed by Matson *et al.*, 2006). The selenoenzyme thioredoxin reductase influences the regulation and expression of genes involved in the innate and adaptive immune responses (reviewed by Maggini *et al.*, 2007). It is possible that exposure to slightly elevated concentrations of Se enhanced the expression of genes associated with innate immunity and enhanced bactericidal capacity. In fish fed supplemental Se, lysozyme activity is enhanced (Lin and Shiau, 2007), and this may have contributed to the enhancement of bactericidal capacity that we detected.

Overall, our results indicated that nestling tree swallows near the spill site were exposed to modest increases in element concentrations from a recently remediated fly ash spill. Exposure to low element concentrations was largely unrelated to several aspects of physiology in nestling tree swallows. It is currently unknown whether exposure to elements during development has long-term effects on physiology in adulthood or how chronic exposure to low element concentration may be related to physiology. Future studies should address the long-term effects of element exposure on physiology, particularly aspects of physiology that could ultimately affect recruitment and survival of young or future reproductive success. Additionally, concentrations of similar combinations of elements would be much higher in active fly ash settling basins (Bryan *et al.*, 2012), potentially putting swallows and other taxa attracted to these sites at greater risk of exposure and physiological effects. In the near future, concentrations of Hg and other heavy metals in fly ash are expected to increase as new clean air regulations reduce air emissions by coal-burning power plants and increase the concentration of these elements in the solid waste stream (USEPA, 2012). Thus, the hazards posed by fly ash are projected to increase in the future, warranting disposal procedures that minimize its potential to contaminate ground and surface water in order to prevent exposure and adverse effects in wildlife.

## References

[1] Acevedo-Whitehouse K Duffus ALJ (2009) Effects of environmental change on wildlife health. Phil Trans R Soc Lond B Biol Sci 364: 3429–3438. 19833653 10.1098/rstb.2009.0128 PMC2781848

[2] Alonso-Alvarez C Tella JL (2001) Effects of experimental food restriction and body-mass changes on the avian T-cell-mediated immune response. Can J Zool 79: 101–105.

[3] ARCADIS (2011) Draft Trace Element Concentrations in Tree Swallows: 2009–2010. TVA, Kingston, TN.

[4] Ardia DR (2005) Super size me: an experimental test of the factors affecting lipid content and the ability of residual body mass to predict lipid stores in nestling European Starlings. Funct Ecol 19: 414–420.

[5] Baos R Blas J Bortolotti GR Marchant TA Hiraldo F (2006a) Adrenocortical response to stress and thyroid hormone status in free-living nestling white storks (*Ciconia ciconia*) exposed to heavy metal and arsenic contamination. Environ Health Perspect 114: 1497–1501. 17035132 10.1289/ehp.9099 PMC1626439

[6] Baos R Jovani R Forero MG Tella JL Gomez G Jimenez B Gonzalez MJ Hiraldo F (2006b) Relationships between T-cell-mediated immune response and Pb, Zn, Cu, Cd, and As concentrations in blood of nestling white storks (*Ciconia ciconia*) and black kites (*Milvus migrans*) from Donana (southwestern Spain) after the Aznalcollar toxic spill. Environ Toxicol Chem 25: 1153–1159. 16629156 10.1897/05-395r.1

[7] Baos R Jovani R Serrano D Tella JL Hiraldo F (2012) Developmental exposure to a toxic spill compromises long-term reproductive performance in a wild, long-lived bird: the White Stork (*Ciconia ciconia*). PLoS One 7: pe34716. 10.1371/journal.pone.0034716 PMC3329485 22529928

[8] Beck ML Hopkins WA Jackson BP (2013) Spatial and temporal variation in the diet of tree swallows: implications for trace element exposure following habitat remediation. Arch Environ Contam Toxicol 65: 575–587. 23695717 10.1007/s00244-013-9913-5

[9] Bichet C Scheifler R Coeurdassier M Julliard R Sorci G Loiseau C (2013) Urbanization, trace metal pollution, and malaria prevalence in the house sparrow. PLoS One 8: pe53866. 10.1371/journal.pone.0053866 PMC3547037 23342022

[10] Biser JA Vogel LA Berger J Hjelle B Loew SS (2004) Effects of heavy metals on immunocompetence of white-footed mice (*Peromyscus leucopus*). J Wildl Dis 40: 173–184. 15362816 10.7589/0090-3558-40.2.173

[11] Blakley BR Sisodia CS Mukkur TK (1980) Effect of methylmercury, tetraethyl lead, and sodium arsenite on the humoral immune-response in mice. Toxicol Appl Pharmacol 52: 245–254. 6892663 10.1016/0041-008x(80)90111-8

[12] Brady C Petrie S Schummer M Badzinski S Belzile N Chen Y-W (2013) Effects of dietary selenium on the health and survival of captive wintering lesser scaup. Environ Pollut 175: 8–15. 23313732 10.1016/j.envpol.2012.12.005

[13] Brasso RL Cristol DA (2008) Effects of mercury exposure on the reproductive success of tree swallows (*Tachycineta bicolor*). Ecotoxicology 17: 133–141. 17701345 10.1007/s10646-007-0163-z

[14] Bryan AL Hopkins WA Parkikh JH Jackson BP Unrine JM (2012) Coal fly ash basins as an attractive nuisance to birds: parental provisioning exposes nestlings to harmful trace elements. Environ Pollut 161: 170–177. 22230082 10.1016/j.envpol.2011.10.021

[15] Cook RB Suter GW Sain ER (1999) Ecological risk assessment in a large river-reservoir: 1. Introduction and background. Environ Toxicol Chem 18: 581–588.

[16] Custer CM (2011) Swallows as sentinel species for contaminant exposure and effect studies. In Elliott J Bishop C Morrissey C, eds, Wildlife Ecotoxicology: Forensic Approaches. Springer, New York, NY, pp 45–91.

[17] Custer CM Gray BR Custer TW (2010) Effects of egg order on organic and inorganic element concentrations and egg characteristics in tree swallows, *Tachycineta bicolor*. Environ Toxicol Chem 29: 909–921. 20821521 10.1002/etc.88

[18] Dan G Lall SB Rao DN (2000) Humoral and cell mediated immune response to cadmium in mice. Drug Chem Toxicol 23: 349–360. 10826101 10.1081/dct-100100120

[19] Dauwe T Janssens E Pinxten R Eens M (2005) The reproductive success and quality of blue tits (*Parus caeruleus*) in a heavy metal pollution gradient. Environ Pollut 136: 243–251. 15840532 10.1016/j.envpol.2005.01.009

[20] Eeva T Hasselquist D Langefors X Tummeleht L Nikinmaa M Ilmonen P (2005) Pollution related effects on immune function and stress in a free-living population of pied flycatcher *Ficedula hypoleuca*. J Avian Biol 36: 405–412.

[21] EPA (1996) Method 3050B Acid digestion of sediments, sludges, and soils. United States Environmental Protection Agency, http://www.epa.gov/osw/hazard/testmethods/sw846/pdfs/3050b.pdf

[22] EPA (2008) Method 6020A Inductively Coupled Plasma-Mass Spectrometry. United States Environmental Protection Agency, http://www.epa.gov/osw/hazard/testmethods/sw846/pdfs/6020a.pdf

[23] Franceschini MD Custer CM Custer TW Reed JM Romero LM (2008) Corticosterone stress response in tree swallows nesting near polychlorinated biphenyl- and dioxin-contaminated rivers. Environ Toxicol Chem 27: 2326–2331. 18476749 10.1897/07-602.1

[24] Franceschini MD Lane OP Evers DC Reed JM Hoskins B Romero LM (2009) The corticosterone stress response and mercury contamination in free-living tree swallows, *Tachycineta bicolor*. Ecotoxicology 18: 514–521. 19360470 10.1007/s10646-009-0309-2

[25] Franson JC Hoffman DJ Schmutz JA (2002) Blood selenium concentrations and enzyme activities related to glutathione metabolism in wild emperor geese. Environ Toxicol Chem 21: 2179–2184. 12371495

[26] Franson JC Hoffman DJ Wells-Berlin A Perry MC Shearn-Bochsler V Finley DL Flint PL Hollmén T (2007) Effects of dietary selenium on tissue concentrations, pathology, oxidative stress, and immune function in common eiders (*Somateria mollissima*). J Toxicol Environ Health A 70: 861–874. 17454562 10.1080/15287390701212760

[27] Gagnon A Jumarie C Hontela A (2006) Effects of Cu on plasma cortisol and cortisol secretion by adrenocortical cells of rainbow trout (*Oncorhynchus mykiss*). Aquat Toxicol 78: 59–65. 16564581 10.1016/j.aquatox.2006.02.004

[28] Green AJ (2001) Mass/length residuals: measures of body condition or generators of spurious results? Ecology 82: 1473–1483.

[29] Handy RD (2003) Chronic effects of copper exposure versus endocrine toxicicty: two side of the same toxicological process? Comp Biochem Physiol A Mol Integr Physiol 135: 25–38. 12727547 10.1016/s1095-6433(03)00018-7

[30] Hawley DM Hallinger KK Cristol DA (2009) Compromised immune competence in free-living tree swallows exposed to mercury. Ecotoxicology 18: 499–503. 19322655 10.1007/s10646-009-0307-4

[31] Heinz GH (1996) Selenium in birds. In Beyer WN Heinz GH Redmon-Norwood AW, eds, Environmental Contaminants in Wildlife: Interpreting Tissue Concentrations. CRC Lewis, Boca Raton, FL, pp 447–458.

[32] Heinz GH Fitzgerald MA (1993) Overwinter survival of mallards fed selenium. Arch Environ Contam Toxicol 25: 90–94.

[33] Herring G Ackerman JT Herzog MP (2012) Mercury exposure may suppress baseline corticosterone levels in juvenile birds. Environ Sci Technol 46: 6339–6346. 22578153 10.1021/es300668c

[34] Hopkins WA Mendonça MT Congdon JD (1997) Increased circulating levels of testosterone and corticosterone in southern toads, *Bufo terrestris*, exposed to coal combustion waste. Gen Comp Endocrinol 108: 237–246. 9356219 10.1006/gcen.1997.6969

[35] Janssens E Dauwe T Bervoets L Eens M (2001) Heavy metals and selenium in feathers of great tits (*Parus major*) along a pollution gradient. Environ Toxicol Chem 20: 2815–2820. 11764165

[36] Janz DM DeForest DK Brooks ML Chapman PM Gilron G Hoff D Hopkins WA McIntyre DO Mebane CA Palace VP (2010) Selenium toxicity to aquatic organisms. In Chapman PM Adams WJ Brooks ML Delos CG Luoma SN Maher WA Ohlendorf HM Presser TS Shaw DP, eds, Ecological Assessment of Selenium in the Aquatic Environment. CRC Press, New York, NY, pp 141–231.

[37] Kloet Ed (1991) Brain corticosteroid receptor balance and homeostatic control. Front Neuroendocrinol 12: 95–164.

[38] Koivula MJ Eeva T (2010) Metal-related oxidative stress in birds. Environ Pollut 158: 2359–2370. 20382455 10.1016/j.envpol.2010.03.013

[39] Koller LD (1973) Immunosuppression produced by lead, cadmium, and mercury. Am J Vet Res 34: 1457–1458. 4748731

[40] Landys MM Ramenofsky M Wingfield JC (2006) Actions of glucocorticoids at a seasonal baseline as compared to stress-related levels in the regulation of periodic life processes. Gen Comp Endocrinol 148: 132–149. 16624311 10.1016/j.ygcen.2006.02.013

[41] Liebl AL Martin LB (2009) Simple quantification of blood and plasma antimicrobial capacity using spectrophotometry. Funct Ecol 23: 1091–1096.

[42] Lifjeld JT Dunn PO Whittingham LA (2002) Short-term fluctuations in cellular immunity of tree swallows feeding nestlings. Oecologia 130: 185–190. 10.1007/s004420100798 28547140

[43] Lin YH Shiau SY (2007) The effects of dietary selenium on the oxidative stress of grouper, *Epinephelus malabaricus*, fed high copper. Aquaculture 267: 38–43.

[44] Maggini S Wintergerst ES Beveridge S Hornig DH (2007) Selected vitamins and trace elements support immune function by strengthening epithelial barriers and cellular and humoral immune responses. Br J Nutr 98: S29–S35. 17922955 10.1017/S0007114507832971

[45] Markman S Mueller CT Pascoe D Dawson A Buchanan KL (2011) Pollutants affect development in nestling starlings *Sturnus vulgaris*. J Appl Ecol 48: 391–397.

[46] Marmiroli N Maestri E (2008) Health implications of trace elements in the environment and the food chain. In Prasad MNV, ed, Trace Elements as Contaminants and Nutrients. John Wiley and Sons, Hoboken, NJ, pp 23–49.

[47] Martin LB Han P Lewittes J Kuhlman JR Klasing KC Wikelski M (2006) Phytohemagglutinin-induced skin swelling in birds: histological support for a classic immunoecological technique. Funct Ecol 20: 290–299.

[48] Martin LB Hopkins WA Mydlarz LD Rohr JR (2010) The effects of anthropogenic global changes on immune functions and disease resistance. Ann N Y Acad Sci 1195: 129–148. 20536821 10.1111/j.1749-6632.2010.05454.x

[49] Massányi P Bárdos L Oppel K Hluchý S Kovácik J Csicsai G Toman R (1999) Distribution of cadmium in selected organs of mice: effects of cadmium on organ contents of retinoids and beta-carotene. Acta Physiol Hung 86: 99–104. 10741868

[50] Matson KD Tieleman BI Klasing KC (2006) Capture stress and the bactericidal competence of blood and plasma in five species of tropical birds. Physiol Biochem Zool 79: 556–564. 16691521 10.1086/501057

[51] NRC (2006) Managing Coal Combustion Residues in Mines. National Research Council, National Academies Press, Washington, DC.

[52] Nyholm NEI (1998) Influence of heavy metal exposure during different phases of the ontogeny on the development of pied flycatchers, Ficedula hypoleuca, in natural populations. Arch Environ Contam Toxicol 35: 632–637. 9776781 10.1007/s002449900425

[53] Ohlendorf HM (2003)Ecotoxicology of selenium. In Hoffman DJ Rattner BA Burton GAJ Cairns JJ, eds, Handbook of Ecotoxicology, Ed 2 CRC Press, Boca Raton, FL, pp 465–500.

[54] Ohlendorf HM Heinz GH (2011) Selenium in birds. In Beyer WN Meador JP, eds, Environmental Contaminants in Biota. CRC Press, Boca Raton, FL, pp 669–701.

[55] Ohlendorf HM Hoffman DJ Saiki MK Aldrich TW (1986) Embryonic mortality and abnormalities of aquatic birds: apparent impacts of selenium from irrigation drainwater. Sci Total Environ 52: 49–63.

[56] Orłowski G Kasprzykowski Z Dobicki W Pokorny P Polechoński R (2010) Geographical and habitat differences in concentrations of copper, zinc and arsenic in eggshells of the Rook *Corvus frugilegus* in Poland. J Ornithol 151: 279–286.

[57] Palacios MG Cunnick JE Winkler DW Vleck CM (2012) Interrelations among immune defense indexes reflect major components of the immune system in a free-living vertebrate. Physiol Biochem Zool 85: 1–10. 22237284 10.1086/663311

[58] Peretz A (1989) Selenium in inflammation and immunity. Proceedings of the 2nd International Congress on Trace Elements in Medicine and Biology, Avoriaz, France, March 1988. In Neve J Favier A, eds, Selenium in Medicine and Biology. de Gruyter, Berlin, pp 235–246.

[59] Pollock B Machin KL (2009) Corticosterone in relation to tissue cadmium, mercury and selenium concentrations and social status of male lesser scaup (*Aythya affinis*). Ecotoxicology 18: 5–14. 18677562 10.1007/s10646-008-0250-9

[60] Ponton F Wilson K Holmes AJ Cotter SC Raubenheimer D Simpson SJ (2013) Integrating nutrition and immunology: a new frontier. J Insect Physiol 59: 130–137. 23159523 10.1016/j.jinsphys.2012.10.011

[61] Potmis RA Nonavinakere VK Rasekh HR Early JL 2nd (1993) Effect of selenium (Se) on plasma ACTH, beta-endorphin, corticosterone and glucose in rat: influence of adrenal enucleation and metyrapone pretreatment. Toxicology 79: 1–9. 8386402 10.1016/0300-483x(93)90201-3

[62] Reilly C (2006) Selenium in Food and Health. Springer, New York, NY.

[63] Ritchie BW Harrison GJ Harrison LR (1994) Avian Medicine: Principles and Application. Wingers Publishing, Lake Worth, FL.

[64] Robertson RJ Stutchbury BJ Cohen RR Winkler DW Hallinger KK Ardia DR (2011) Tree Swallow. The Birds of North America-On-Line, http://bna.birds.cornell.edu/bna/species/011/articles/introduction

[65] Rowe CL Hopkins WA Congdon JD (2002) Ecotoxicological implications of aquatic disposal of coal combustion residues in the United States: a review. Environ Monit Assess 80: 207–276. 12503897 10.1023/a:1021127120575

[66] Sapolsky RM Romero LM Munck AU (2000) How do glucocorticoids influence stress responses? Integrating permissive, suppressive, stimulatory, and preparative actions. Endocr Rev 21: 55–89. 10696570 10.1210/edrv.21.1.0389

[67] Schulte-Hostedde AI Zinner B Millar JS Hickling GJ (2005) Restitution of mass-size residuals: validating body condition indices. Ecology 86: 155–163.

[68] Sherman AR (1992) Zinc, copper, and iron nutriture and immunity. J Nutr 122: 604–609. 1542019 10.1093/jn/122.suppl_3.604

[69] Smits JE Bortolotti GR Tella JL (1999) Simplifying the phytohaemagglutinin skin-testing technique in studies of avian immunocompetence. Funct Ecol 13: 567–572.

[70] Snoeijs T Dauwe T Pinxten R Vandesande F Eens M (2004) Heavy metal exposure affects the humoral immune response in a free-living small songbird, the great tit (*Parus major*). Arch Environ Contam Toxicol 46: 399–404. 15195812 10.1007/s00244-003-2195-6

[71] Snoeijs T Dauwe T Pinxten R Darras VM Arckens L Eens M (2005) The combined effect of lead exposure and high or low dietary calcium on health and immunocompetence in the zebra finch (*Taeniopygia guttata*). Environ Pollut 134: 123–132. 15572230 10.1016/j.envpol.2004.07.009

[72] SPSS (2009) PASW Statistics GradPack 18. SPSS Inc, Chicago, IL.

[73] Stantec (2009) TVA Disposal Facility Assessment Phase 1 Plant Summary Bull Run Fossil Plant (BRF). Nashville, TN, 76 pp.

[74] Surai PF (2006) Selenium in Nutrition and Health. Nottingham University Press, Nottingham, UK.

[75] TVA (2009) Corrective Action Plan for the TVA Kingston Fossil Plant Ash Release. Tennessee Valley Authority, Kingston, TN, 73 pp.

[76] TVA (2011a) TVA Kingston Fossil Fuel Plant Release Site On-Scene Coordinator Report for the Time-Critical Removal Action May 11, 2009 through December 2010. Harriman, TN, 222 pp.

[77] TVA (2011b) Inspection Report: TVA's Groundwater Monitoring at Coal Combustion Products Disposal Areas. Vol. 2009-12991 Office of Inspector General, Tennessee Valley Authority, Knoxville, TN, 29 pp.

[78] USDA (2009) Lower Clinch River rapid watershed assessment. In USDA and Natural Resources Conservation Service, ed, USDA and NRCS, Clinton, TN, 19 pp.

[79] USEPA (2012) National Emission Standards for Hazardous Air Pollutants From Coal- and Oil-Fired Electric Utility Steam Generating Units and Standards of Performance for Fossil-Fuel-Fired Electric Utility, Industrial-Commercial- Institutional, and Small Industrial-Commercial-Institutional Steam Generating Units; Final Rule. Vol 77 National Archives and Records Administration, Washington, DC, pp 9304–9512.

[80] Vitousek PM Mooney HA Lubchenco J Melillo JM (1997) Human domination of Earth's ecosystems. Science 277: 494–499.

[81] Wada H Cristol DA McNabb FMA Hopkins WA (2009) Suppressed adrenocortical resonses and thyroid hormone levels in birds near a mercury-contaminated river. Environ Sci Technol 43: 6031–6038. 19731714 10.1021/es803707f

[82] Wayland M Gilchrist HG Marchant T Keating J Smits JE (2002) Immune function, stress response, and body condition in Arctic-breeding common eiders in relation to cadmium, mercury, and selenium concentrations. Environ Res 90: 47–60. 12359190 10.1006/enrs.2002.4384

[83] Wayland M Smits JEG Gilchrist HG Marchant T Keating J (2003) Biomarker responses in nesting, common eiders in the Canadian Arctic in relation to tissue cadmium, mercury and selenium concentrations. Ecotoxicology 12: 225–237. 12739870 10.1023/a:1022506927708

[84] Weech SA Scheuhammer AM Wayland ME (2012) Selenium accumulation and reproduction in birds breeding downstream of a uranium mill in northern Saskatchewan, Canada. Ecotoxicology 21: 280–288. 21927945 10.1007/s10646-011-0788-9

[85] Wingfield JC Romero LM (2001) Adrenocortical Responses to Stress and Their Modulation in Free-living Vertebrates. Oxford University Press, New York, NY.

[86] Wingfield JC Maney DL Breuner CW Jacobs JD Lynn S Ramenofsky M Richardson RD (1998) Ecological bases of hormone–behavior interactions: the “emergency life history stage”. Am Zool 38: 191–206.

[87] Wintergerst ES Maggini S Hornig DH (2007) Contribution of selected vitamins and trace elements to immune function. Ann Nutr Metab 51: 301–323. 17726308 10.1159/000107673

[88] Yamamoto JT Santolo GM (2000) Body condition effects in American kestrels fed selenomethionine. J Wildl Dis 36: 646–652. 11085425 10.7589/0090-3558-36.4.646

[89] Zwolak I Zaporowska H (2012) Selenium interactions and toxicity: a review. Cell Biol Toxicol 28: 31–46. 21913064 10.1007/s10565-011-9203-9

